# The effect of fish density and tank size on the behavior of adult zebrafish: A systematic analysis

**DOI:** 10.3389/fnbeh.2022.934809

**Published:** 2022-10-05

**Authors:** Stephanie Shishis, Benjamin Tsang, Robert Gerlai

**Affiliations:** ^1^Department of Cell & Systems Biology, University of Toronto, Toronto, ON, Canada; ^2^Department of Psychology, University of Toronto Mississauga, Mississauga, ON, Canada; ^3^Department of Critical Care Medicine, Hospital for Sick Children, Toronto, ON, Canada

**Keywords:** psychological stress, physiological stress, crowding, anxiety, laboratory housing

## Abstract

The zebrafish has been employed in several fields of biology due to its translational relevance and its simplicity and ease of maintenance. As a result, zebrafish are kept in thousands of laboratories around the world. Current industry standards favor keeping the largest possible number of fish in the smallest possible volume of water to increase efficiency and reduce costs. However, physiological and psychological stress resulting from such crowding may impact a variety of phenotypes, from brain function and behavior to cardiovascular function and cancer. Nevertheless, surprisingly little is known about what constitutes an optimal housing environment for the zebrafish, e.g., no systematic analyses have been performed to test the role of housing density and tank volume despite recent sporadic reports implying negative effects of the standard practice of crowding. Here, we conduct the first proof of concept analysis examining the potential impact of housing density and tank volume on the behavior of zebrafish. We randomly assigned adult zebrafish to one of three tank sizes (1.5, 10, or 50 L) with one of three housing densities (1, 2, or 4 fish/L), a 3 × 3 between subject experimental design, and maintained the fish in their corresponding condition for 2 weeks. Subsequently, we tested the behavior of the fish singly in a novel open tank for 12 min and quantified several of their swim path parameters using a video-tracking system. We found significant additive and interacting effects of tank size and/or housing density on swim path parameters including immobility, swim speed, turn angle, and distance to bottom and to stimulus. Although we had only three fish densities and three tank sizes and we did not explore the effects of more extreme conditions and although the interpretation of the above behavioral effects is speculative at this point, the results already demonstrate that both tank size and housing density exerts significant effects on the zebrafish and thus should be considered in zebrafish husbandry.

## Introduction

The zebrafish (*Danio rerio*) is a freshwater fish found in slow flowing bodies of water such as ponds and streams in Southern and South-Eastern Asia (Reed and Jennings, 2011). It has become one of the most favored laboratory model organism in biomedical research (Parker et al., 2012; Shams et al., 2017a,b). The zebrafish is a simple vertebrate that possesses a sophisticated behavioral repertoire and well characterized biological features including thoroughly investigated embryonic development (Luca and Gerlai, 2012; Fulcher et al., 2017; Shams et al., 2017b). The zebrafish genome is sequenced with a number of genes identified as having homologs in mammals, and numerous other features of this species, including neurobiological and neuroanatomical characteristics have also been found to be evolutionarily conserved when compared to mammalian phenes (Luca and Gerlai, 2012; Fulcher et al., 2017; Shams et al., 2017a,b). Due to its simplicity, its economical maintenance, and its prolific nature, the zebrafish is employed increasingly frequently in a variety of fields of biology including behavioral neuroscience (Luca and Gerlai, 2012; Fulcher et al., 2017; Shams et al., 2017a,b). As a result, thousands of laboratories keep zebrafish around the world.

In most zebrafish facilities, the fish are housed in commercial high-density rack systems that employ efficient recirculating multi-stage water filtration (Avdesh et al., 2012; Lawrence and Mason, 2012). The tank sizes on these high-density rack systems range from 1 to 10 L in volume, and the excellent filtration methodology allows housing zebrafish at a high density of 5–20 fish/L (Avdesh et al., 2012; Lawrence and Mason, 2012; Liss et al., 2015; Aleström et al., 2019). The high-density housing method reduces costs and increases maintenance efficiency for the zebrafish researcher. In the past, the primary concern for most researchers studying embryonic development was egg-yield, and thus efficiency and cost of fish breeding and maintenance enjoyed priority. However, with the increasingly sophisticated methods employed in current zebrafish biology research and the expected finer phenotypical changes resulting from a variety of manipulations, including those affecting brain function and behavior, zebrafish researchers need to revisit the question of whether the current industry standard of crowding zebrafish into small tanks may impact the studied phenotypes. Sporadic studies have already started to imply that housing zebrafish in the standard manner may have deleterious consequences (Ramsay et al., 2006; Reed and Jennings, 2011; Parker et al., 2012; Pavlidis et al., 2013; Lindsey and Tropepe, 2014; Shams et al., 2015, 2017a,b; Movva et al., 2017; Maierdiyali et al., 2020; Stevens et al., 2021). Surprisingly, despite the increasing popularity and frequent use of zebrafish in biomedical research, little is known about what constitutes an optimal housing environment for these fish in the laboratory, and no systematic analysis has been performed to address this question (Ramsay et al., 2006; Reed and Jennings, 2011; Andersson and Kettunen, 2021).

The zebrafish is a highly social species. In their natural environment, these fish form shoals ranging from two to several hundred individuals, an important adaptive strategy that reduces predation risk and increases foraging efficiency and mating success (Miller and Gerlai, 2011; Shams et al., 2015, 2017a,b; Movva et al., 2017; Tunbak et al., 2020; Stevens et al., 2021). Although zebrafish are highly social and shoal in the laboratory too, these fish do not live in confined, cave-like environments, but inhabit open, spacious bodies of water (Ramsay et al., 2006; Reed and Jennings, 2011). Thus, housing zebrafish in physically confining small tanks may be highly unnatural. Housing zebrafish in small tanks with high density may induce psychological as well as physiological stress. For example, psychological stress may result from increased competition for food and from elevated the frequency of agonistic encounters, or from the fact that subordinate, weaker or smaller fish will be unable to escape from larger, stronger dominant ones. Physiological stress may result from reduced oxygen levels as large number of fish crowded into a small tank may deplete oxygen. Reduction of oxygen levels may also result from accumulation of organic waste, which again is expected to be larger in small tanks crowded with fish. Furthermore, crowding fish into small tanks may also increase time-dependent fluctuations in the level of organic waste products. This is expected because the central filtration system cannot remove locally produced organic waste that happens in case of overfeeding or when the large number of fish fed produce faces. These changes occur relatively fast in individual tanks undetected by the central monitoring equipment of zebrafish high-density racks, and can lead to rapid short-term elevation of toxin levels in the given tank especially if it is small in volume and stocked with a large number of fish.

Indeed, recent studies have already begun to imply that the current method of housing zebrafish in large numbers in small tanks is suboptimal likely leading to conditions that may negatively impact a plethora of biological phenotypes including brain function and behavior (Ramsay et al., 2006; Reed and Jennings, 2011; Parker et al., 2012; Pavlidis et al., 2013; Lindsey and Tropepe, 2014; Shams et al., 2015, 2017a,b; Movva et al., 2017; Maierdiyali et al., 2020; Stevens et al., 2021). For example, unexpectedly, housing the highly social zebrafish singly was found to reduce anxiety-like responses (e.g., hyperactivity and thigmotaxis) compared to the behavior of zebrafish housed in a standard crowded manner (Shams et al., 2017a). Furthermore, paradoxically, whole-body cortisol levels were also found reduced in chronically isolated fish, a change that was accompanied by reduced bottom dwell time, a behavioral sign of diminished anxiety (Shams et al., 2015, 2017b). These results suggested that zebrafish forced to be in dense groups in small tanks may experience elevated stress and/or anxiety (Parker et al., 2012; Movva et al., 2017). Lower cortisol release rates were also demonstrated in group-housed zebrafish that were exposed to short term (1 h) or long-term (2 weeks) social isolation as compared to group-housed controls (Lindsey and Tropepe, 2014). Similarly, when zebrafish were housed in lager groups versus housed as pairs, the former showed behavioral signs of elevated anxiety including increased diving, meandering, bottom dwelling and angular velocity (Movva et al., 2017; Stevens et al., 2021). In accordance with these findings, whole-body cortisol levels of zebrafish housed in crowded (40 fish/L) versus non-crowded (0.25 fish/L) tanks were found to be significantly elevated in the former (Ramsay et al., 2006). Similarly, zebrafish housed at a density of 5 fish/L were found to have significantly lower cortisol release rates than those housed at densities of 10, 20, or 40 fish/L, respectively (Pavlidis et al., 2013). The effect of tank size has also started to be explored (Maierdiyali et al., 2020). Shelter leaving/shelter seeking and shoaling responses as well as stamina were found to be negatively affected in zebrafish housed in small tanks compared to those housed in large tanks, suggesting increased shyness, reduced social behavior and poorer stamina in the former (Maierdiyali et al., 2020).

Although the above reviewed sporadic studies all suggest that housing zebrafish at high density in small tanks may be suboptimal (Ramsay et al., 2006; Reed and Jennings, 2011; Parker et al., 2012; Pavlidis et al., 2013; Lindsey and Tropepe, 2014; Shams et al., 2015, 2017a,b; Movva et al., 2017; Maierdiyali et al., 2020; Stevens et al., 2021), systematic analyses of the effects of housing density and housing tank size have not been performed. In the current study, we employ three housing densities (fish density) and three home tank sizes (housing tank size) using a 3 × 3 between subject design, exposing our experimental fish to their respective housing condition for 2 weeks. We treat these two factors (fish density and housing tank size) in our analyses as independent variables and ask whether they affect the behavior of zebrafish in an additive or interactive manner. As a readout of our treatments, we quantify the behavior of the experimental fish in a novel tank exploration task. We chose behavior as the endpoint of our analysis because we argue behavioral analysis allows us to detect the potential phenotype altering effects of housing conditions in a simple and unbiased manner. In sum, we predict that higher housing density especially in small tanks have negative consequences and will induce stress and anxiety that will manifest as altered behavioral responses of the experimental zebrafish to a mildly aversive novel tank.

Here we report significant behavioral effects of both fish density and housing tank size. However, we regard these results only as proof-of-concept, as we acknowledge that more extreme conditions may need to be tested and a larger number of levels of our two factors must be employed in future systematic analyses in order for one to find the most optimal housing conditions, and also because detailed follow up behavioral, neurochemical and neurohormonal analyses may be required to properly interpret the behavioral results.

## Materials and methods

### Housing and husbandry

For this study, adult wildtype zebrafish (*Danio rerio*) were bred in our facility (the Gerlai Zebrafish Facility of the University of Toronto Mississauga). These fish originated from a local pet store (Big Al’s Aquarium warehouse, Mississauga, ON, Canada), which obtains their stock from large scale breeding facilities in Asia. This population of zebrafish is expected to be genetically heterogeneous (high genetic variance in the population) with individuals having the majority of their genetic loci heterozygous. We chose this population because unlike inbred and quasi-inbred strains, which are expected to be genetically unique, our wildtype genetically heterogeneous population is expected to possess fewer idiosyncratic features, and thus better represent species-typical characteristics of the zebrafish. We bred and raised our fish in our facility as described before (Abozaid et al., 2020; Facciol et al., 2022). Briefly, fry from multiple breeding pairs were pooled and raised in 10 L housing tanks placed on the Aquaneering (San Diego, CA, USA) system racks until they were randomly selected and transferred to their respective experimental housing tank at their age of 3 months (see below). All experiments/procedures were approved by the Local Animal Care Committee and were in accordance with guidelines set by the Canadian Council on Animal Care. All experimental fish were housed in tanks that were part of recirculating Aquaneering (San Diego, CA, USA) aquatic housing system racks. These racks were equipped with biological, chemical, and mechanical filtration in addition to UV sterilization. Tanks received the same level of filtration (volume/time filtered water). Water temperature, salinity and pH in the housing tanks were maintained at 26–27°C, 150–300 μS, and 6.5–8.0, respectively. The light cycle in the facility was 12 h light:12 h dark with lights gradually turning on at 6:30 h and off at 21:00 h *via* a computer-controlled dimmer. All fish were fed twice a day, between 11:30 and 12:30, and between 14:00 and 16:00 using a combination of freshly hatched nauplii of brine shrimp (A. salina, Brine Shrimp Direct, Ogden, UT, USA) and dried zebrafish micro-pellets (Zeigler Bros, Inc., Gardners, PA, USA).

### Experimental design

Upon reaching 3 months post-fertilization, zebrafish were randomly assigned to nine groups. The experimental design was a two-factorial between subject design with independent between-subject factors, tank size (3 levels) and fish density (3 levels), giving 3 × 3 = 9 treatment conditions (groups) with no replicates. The three housing tank sizes were 1.5 L (length × width × height: 26.5 cm × 5.5 cm × 15 cm), 10 L (length × width × height: 33 cm × 21 cm × 19 cm), and 50 L (length × width × height: 68.5 cm × 33 cm × 22 cm), and the three housing densities (fish densities) were 1, 2, and 4 fish/L. These conditions were chosen as they represent the usual housing parameters several zebrafish facilities employ. We note that some facilities employ much higher fish densities with likely more severe effects. However, in the current study we decided to avoid such high densities, and we did not employ larger number of levels of fish density and tank size as we only wanted to demonstrate first that these factors have an effect on the behavior of zebrafish. We also note that the smallest tank and lowest fish density meant that the experimental fish were housed singly. This is a unique condition for the highly social zebrafish, but one which is often employed in numerous studies, including those that investigate learning and memory and require identification of individuals without the use of invasive marking techniques for multiple consecutive trials performed over days or weeks (Gerlai, 2017, 2016). We also note that isolation is often employed for other purposes, including for the analysis of anxiety and stress (Shams et al., 2017a,b).

The experimental zebrafish were transferred into their above-described corresponding experimental housing tanks where they remained for 2 weeks, a chronic housing condition treatment. Sexes were housed mixed, and the sex ratio was approximately 50:50% in all housing tanks. To block visual cues and interaction between fish from neighboring tanks, white paper dividers in clear plastic sleeves were placed in between the housing tanks. After the 2-week long housing condition treatment, all fish underwent behavioral testing. The rationale behind this was that it included human handling and placing the fish into a novel tank, procedures that are routinely performed in most zebrafish studies, including behavioral neuroscience experiments. The behavioral testing was conducted in a dedicated testing room that was adjacent to the housing room in the Facility. All behavioral tests occurred between 9:00 and 16:00 h. Each experimental fish was tested once, and every day one fish per experimental group was tested in an order randomized across the housing conditions. Approximately 19 fish were tested per condition (nine conditions), i.e., a total of 169 fish were analyzed (for exact sample sizes, see Table 1).

**TABLE 1:** Sample sizes (*n*) for the nine experimental treatment groups defined by housing conditions, tank size and fish density.

		Tank size (L)		
		1.5	10	50
**Fish density (#/L)**	1	19	19	20
	2	18	17	19
	4	20	19	18

### Behavioral apparatus

The behavioral test was conducted in a 40 L experimental tank (50 cm × 25 cm × 30 cm, length × width × depth) placed on a shelf in a dedicated behavioral test room where experimenters did not enter during the behavioral test sessions. The bottom and back of the experimental tank were covered with white Bristol Board. An LCD computer monitor (Acer Model No. EB210HQ) was placed flush on the two sides of the tank. A digital HD video camera (JVC GZ-MG330HV) was positioned in front of the experimental tank. Behind the camera, the experimental fish could only see a light-green painted wall with no other stimuli. Water parameters in the experimental tank were maintained identical to those of the housing tanks. Water of the experimental tank was changed every morning before testing. Furthermore, to equalize the amount of olfactory cues present in the water of the experimental tank in the morning versus the afternoon, 10 stimulus zebrafish were placed in the experimental tank for 12 min and subsequently removed, before behavioral testing of experimental zebrafish commenced. These stimulus fish were of identical size and population origin as the test fish and were also housed in an identical manner.

### Testing procedure

For each behavioral recording session, a single experimental fish was transferred from its housing tank to the testing tank. The fish was gently netted, quickly placed into a carrying beaker, transferred to the adjacent behavioral testing room, and then gently decanted from the holding beaker into the test tank. Recording of the behavior of the experimental zebrafish started immediately after the fish was placed into the test tank. During the test visual stimuli were also presented at given time points using the computer monitors that flanked the test tank. These stimuli were supposed to mimic some aspects of the natural environment of the zebrafish. For example, at 300th s a group of 10 still zebrafish images of the same length as that of the test subject was presented in the middle of one of the monitors *via* PowerPoint for 1 min (300–360 s) to mimic the appearance of a shoal (Fernandes and Gerlai, 2009; Qin et al., 2014). At the 420th s, using Powerpoint, an expanding black dot was presented for 7.5 s in the middle of the monitor that previously delivered. The expanding dot is expected to mimic the rapid frontal approach of a piscivore and have been found to be aversive for zebrafish (Luca and Gerlai, 2012). A set of zebrafish images were again shown for 1 min (457.5–517.5 s) on the opposite monitor, and an expanding black dot was shown on this latter monitor again for 7.5 s (577.5–585 s). In between the stimulus presentation periods the monitors were showing a white blank screen. The starting position of the stimulus presentation side varied randomly among experimental fish. A similar aversive and appetitive stimulus presentation regimen was first employed by Fernandes and Gerlai (2009). Recording of the behavior of the experimental zebrafish continued throughout the entire 12 min session. At the conclusion of the behavioral test, the experimental fish was gently removed from the test tank and sacrificed immediately for later whole-body cortisol and neurochemical analysis (Tran et al., 2014).

### Behavioral measures and data extraction

Video recordings saved by the digital video camera were transferred to a hard drive of a computer and later analyzed using the Noldus Ethovision Color Pro 12 (Noldus InfoTech., Wageningen, Netherlands) tracking software. Center-point detection was used for tracking. The testing arena was calibrated to scale. The tracking system used reference images from prior video files for detection. We employed dynamic subtraction for subject detection where the range of contrast (bright and dark) between subject and background as well as frame weight were assessed. Video pixel smoothing was not used, but track noise reduction was employed. To remove indentations in the shape of the subject or effects of stripes on the body of the fish to give a smoother outline and ensure that Ethovision detected the subject as one animal, the subject contour was set to dilate first, then erode. The subject size (surface area in video pixels) was set to a minimum threshold of 0 and maximum threshold of 800–900 to prevent objects including reflections from being detected during tracking.

Numerous swim path parameters were extracted as follows. Duration of immobility (sec) is quantified by Ethovision when the number of pixels corresponding to the total visible surface area of the experimental subject changing from one video-frame to the next was below 20% (with 30 frame per sec temporal resolution). Speed (cm/sec) of swimming is measured as the velocity of the center point of the fish. Variance of speed (cm^2^/sec^2^) measures the intra-individual temporal variance of velocity, i.e., whether the given experimental fish swims with consistent speed (low variance) or varying speeds (high variance). Absolute turn angle (degree) quantifies the change in the direction of movement (turning) irrespective of whether it is clockwise or counter-clockwise. Turn angle variance (degree 2) quantifies the intra-individual temporal variance of turning, i.e., measures whether the test subject turned with high consistency (low variance) or sometimes by a great degree other times by a small degree (high variance). Duration of high mobility quantifies the period of time during which the change in the total number of pixels corresponding to the detected experimental fish exceeded 60% from one video-frame to the next (with 30 frame per s temporal resolution). Distance to bottom (cm) measures how far the fish swam relative to the bottom of the test tank. Variance of distance to bottom quantifies the intra-individual temporal variance of distance to the bottom (vertical exploration), i.e., measures whether the test fish stayed at a consistent distance to the bottom (low variance) or varied its location on the vertical axis a lot (high variance). The last parameter we extracted was the distance to stimulus side, which measures how far the test fish stayed relative to the computer monitor that was presenting or just presented the shoaling or the expanding dot stimulus. All the above behavioral parameters have been employed in the analysis of behavioral responses of zebrafish to novel contexts, aversive versus appetitive cues. For example, most of these behaviors have been argued to be a measure of fear and anxiety in zebrafish (Ahmed et al., 2012; Blaser and Rosemberg, 2012; Kalueff et al., 2013; Qin et al., 2014; Gerlai and Tran, 2016; Gerlai, 2020, 2013, 2011, 2010; Ro et al., 2021; Facciol et al., 2022).

### Statistical analysis

The data extracted by the Ethovision tracking software were analyzed using IBM SPSS statistical software (version 24 written for the PC). First, we calculated and expressed the results for 30 s intervals of the 12 min session and analyzed the effect of interval (the within subject repeated measure factor having 24 levels), the effect of housing density (fish density, a between subject factor with 3 levels) and the effect of housing tank size (the second between subject factor with 3 levels) as well as the significance of interaction among these factors using three factorial repeated measures variance analysis (ANOVA). Because *post-hoc* multiple comparison tests are not appropriate for repeated measures designs, and because interval was often found not to interact with the other two factors, we pooled the data for the intervals, and conducted a *post-hoc* non-repeated measures ANOVA (with housing density and housing tank size as the two independent factors) followed by Tukey’s HSD *post-hoc* multiple comparison test, where warranted. The latter test allows one to determine which group is different from which while minimizing type 1 error without committing type 2 error. The method of data pooling for the non-repeated measures ANOVA and subsequent Tukey’s HSD *post-hoc* test depended upon the particular behavioral measure. For example, for duration type measures, including duration of immobility, pooling meant adding the 30 s interval data, i.e., calculating cumulative duration. For other behaviors, including speed, turn angle and distance measures as well as intra-individual temporal variance measures, pooling meant calculating the average of 30 s intervals for the entire session period.

In addition, and because we expected the distance to stimulus measure to be sensitive to the specific presentation periods of the stimuli, we calculated a derived measure for this particular behavior. This measure we call change of distance from habituation period to stimulus presentation period (the habituation period is defined as the entire first part of the behavioral session preceding the first stimulus presentation). We calculated the average of 30 s data for the first 300 s, the habituation period, and subtracted this value from the average of 30 s intervals during which the given stimulus (the expanding dot shown for two 30 s intervals, or the shoaling stimulus shown for four 30 s intervals). For this derived measure, a negative value represents a reduction of distance in response to the stimulus whereas a positive value an increase of distance. We analyzed the change of distance to stimulus using two-factorial non-repeated measures ANOVA and Tukey’s HSD *post-hoc* multiple comparison test. For all statistical analyses, we accepted significance when the probability of the null hypothesis (no effect of an independent factor or interaction, or no difference among groups) was not larger than 5%, i.e., when *p* = < 0.05. We note that all the above parametric statistical procedures are required to meet the criteria for variance homogeneity and normality of distribution. However, we also note that all parametric tests are known to be insensitive to the violation of these criteria if the analyzed groups do not have grossly different sample sizes. In our study, the analyzed groups had practically identical sample sizes.

Subsequent to the univariate statistical analyses, we also conducted a multivariate analysis, Principal Component Analysis, that is based upon bivariate Pearson product moment correlations. This analysis allows one to reduce the complexity of results as it extracts Principal Components that explain the majority of variance in the data and represent correlation groups of behaviors. These correlation groups may reveal behavioral states or strategies (Gerlai and Csányi, 1990). We conducted this analysis using the data pooled for the entire testing period for all behavioral measures including immobility, speed, intra-individual variance of speed, absolute turn angle, intra-individual variance of absolute turn angle, high mobility, distance to bottom, intra-individual variance of distance to bottom, change of distance to shoal image, change of distance to expanding dot. In addition, in this analysis we included the independent treatment factors, tank size and fish density as numerical variables to see how their values correlate with the behavioral responses. That is, we used 12 variables in our analysis. In order for Principal Component Analysis to yield stable and reliable results, one must have at least six times as many subjects as variables (Gerlai and Csányi, 1990). In our study we tested 169 fish and used 12 variables for the Principal Component Analysis, and thus we met this requirement. The Principal Component Analysis was conducted using Varimax Rotation with Kaiser normalization, which creates orthogonal (non-correlating) Principal Components. Principal Components with eigen values reaching 1 were maintained.

## Results

From the swim paths of zebrafish, we first extracted 30 s interval data and investigated whether temporal changes occurred throughout the 12-min long behavioral testing period and whether these changes depended upon the prior housing conditions, i.e., whether they were affected by fish density and/or housing tank size. For almost all behaviors, however, we found that although significant temporal changes occurred, these changes were independent of prior housing conditions. For this reason, we pooled the data for the time intervals and focused our subsequent statistical analysis on behaviors pooled (averaged or summed) for the entire recording period. We also note that pooling the data across intervals allowed us to conduct *post-hoc* multiple comparisons using Tukey’s HSD test without introducing type-1 error, or inflating type-2 error. The detailed results of our statistical analyses are shown in Table 2. Below we summarize the results separately for each behavioral measure.

**TABLE 2:** Results of ANOVAs.

Results of analysis of variance							
Between subject effects				Within subject effects			
Behavior	Fish density	Tank size	Fish density × Tank size	Interval	Interval × Fish density	Interval × Tank size	Interval × Fish density × Tank size
Speed	*F*_(2, 160)_ = 0.232, *p* = 0.793	*F*_(2, 160)_ = 1.674, *p* = 0.191	***F***_(4, 160)_ **= 2.829, *p* = 0.027**	***F***_(23, 3680)_ **= 7.208, *p* < 0.001**	*F*_(46, 3680)_ = 0.727, *p* = 0.916	*F*_(46, 3680)_ = 1.074, *p* = 0.340	*F*_(92, 3680)_ = 0.822, *p* = 0.812
Duration of Immobility	*F*_(2,160)_ = 0.855, *p* = **0.427**	***F***_(2,160)_ **= 13.596, *p* = 0.000**	***F***_(4,160)_ **= 8.076, *p* = 0.000**	***F***_(23,3680)_ **= 3.021, *p* = 0.000**	*F*_(46,3680)_ = 0.727, *p* = 0.896	*F*_(46, 3680)_ = 1.343, *p* = 0.061	*F*_(92,3680)_ = 1.082, *p* = 0.281
Variance of speed	***F***_(2,160)_ **= 17.033, *p* < 0.001**	***F***_(2,160)_ **= 11.904, *p* < 0.001**	***F***_(4,160)_ **= 5.544, *p* < 0.001**	***F***_(23,3680)_ **= 2.080, *p* = 0.002**	*F*_(46,3680)_ = 1.138, *p* = 0.243	*F*_(46, 3680)_ = 0.589, *p* = 0.988	*F*_(92,3680)_ = 0.912, *p* = 0.713
Absolute turn angle	***F*(2,160) = 25.929, *p* < 0.001**	***F***_(2,160)_ **= 17.253, *p* < 0.001**	***F***_(4,160)_ **= 5.807, *p* < 0.001**	***F***_(23,3680)_ **= 0.813, *p* = 0.718**	*F*_(46,3680)_ = 0.718, *p* = 0.923	*F*_(46, 3680)_ = 1.267, *p* = 0.108	*F*_(92,3680)_ = 0.912, *p* = 0.713
Turn angle variance	***F***_(2,160)_ **= 27.644, *p* < 0.001**	***F***_(2,160)_ **= 21.171, *p* < 0.001**	***F***_(4,160)_ **= 6.968, *p* < 0.001**	*F*_(23,3680)_ = 1.196, *p* = 0.236	*F*_(46,3680)_ = 0.801, *p* = 0.829	*F*_(46, 3680)_ = 1.290, *p* = 0.092	*F*_(92,3680)_ = 1.088, *p* = 0.268
Duration of high mobility	***F***_(2,160)_ **= 4.713, _p_ = 0.010**	***F***_(2,160)_ **= 6.123, *p* = 0.003**	***F***_(4,160)_ **= 5.670, *p* = 0.000**	*F*_(23,3680)_ = 1.626, *p* = 0.030	*F*_(46,3680)_ = 0.801, *p* = 0.830	*F*_(46, 3680)_ = 1.074, *p* = 0.339	*F*_(92,3680)_ = 0.964, *p* = 0.577
Distance to bottom	*F*_(2,160)_ = 0.897, *p* = 0.410	*F*_(2,160)_ = 0.666, *p* = 0.515	***F*(4,160) = 4.529, *p* = 0.002**	***F***_(23,3680)_ **= 12.923, *p* = 0.000**	*F*_(46,3680)_ = 0.733, *p* = 0.910	***F***_(46, 3680)_ **= 1.535, *p* = 0.012**	*F*_(92,3680)_ = 0.949, *p* = 0.619
Variance of distance to bottom	*F*_(2,160)_ = 0.594, *p* = 0.553	*F*_(2,160)_ = 2.539, *p* = 0.082	*F*_(4,160)_ = 2.053, *p* = 0.090	***F***_(23,3680)_ **= 9.262, *p* = 0.000**	*F*_(46,3680)_ = 0.774, *p* = 0.865	***F***_(46, 3680)_ **= 2.255, *p* = 0.000**	*F*_(92,3680)_ = 1.126, *p* = 0.196
Distance to stimulus side	***F***_(2,160)_ **= 4.096, *p* = 0.018**	*F*_(2,160)_ = 0.560, *p* = 0.572	*F*_(4,160)_ = 1.867, *p* = 0.119	***F***_(23,3680)_ **= 1.961, *p* = 0.004**	***F***_(46,3680)_ **= 1.382, *p* = 0.045**	*F*_(46, 3680)_ = 0.712, *p* = 0.928	*F*_(92,3680)_ = 0.980, *p* = 0.534

Significant effects are highlighted by red font in bold typeface.

**FIGURE 1:**
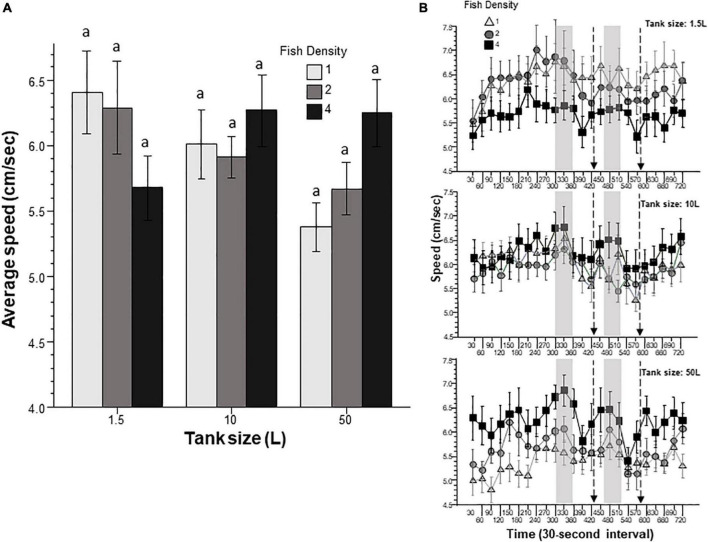
Immobility is affected by prior fish density and home tank size. Panel **(A)** shows the cumulative (total) duration of immobility exhibited by zebrafish during the entire 12 min behavioral test period. The size of the housing tank is shown on the *X*-axis and the fish density is shown by the legend. Bars marked by at least one common letter represent groups that are not different significantly (*p* > 0.05). Note the tank size dependent fish density effects. Panel **(B)** shows the duration of immobility exhibited by the experimental zebrafish as a function of 30-s intervals. The upper line-graph shows the performance of fish housed in 1.5 L tanks, the middle line-graph the performance of fish housed in 10 L tanks and the lower line-graph the performance of fish housed in 50 L tanks. The fish density of fish is shown by the legend. The vertical semi-transparent gray bars indicate the period during which still images of zebrafish were shown on the computer screen, and the broken lines with the arrowhead indicate the short period of administering a computer animated expanding dot. Mean + S.E.M. are shown. For detailed statistical results, see Table 1 and the section “Results.”

Immobility or freezing is often used as an index of fear or anxiety in zebrafish placed in novel tanks, but lack of or reduced activity may also be a sign of habituated state in familiar environments (Cachat et al., 2010; Clark et al., 2011; Blaser and Rosemberg, 2012; Luca and Gerlai, 2012; Kalueff et al., 2013; Gerlai and Tran, 2016). Immobility duration appeared to vary across the nine experimental zebrafish groups (Figure 1). Variance Analysis of the cumulative duration of immobility the fish exhibited during the entire duration of the behavioral test revealed a significant tank size effect as well as a significant fish density x tank size interaction. Tukey’s HSD *post-hoc* analysis also found several group differences detailed in Figure 1A. Perusal of this figure suggests that the interaction was mainly due to the fact that fish housed in the 10 L tanks at the highest density reduced their immobility, but fish housed in the 50 L tanks increased their immobility compared to fish of the other groups, whereas fish housed in the 1.5 L tank were unaffected by prior housing density. Although ANOVA also detected a significant interval effect, the temporal changes did not significantly differ across the nine experimental groups as demonstrated by the lack of interaction between interval and fish density and tank size.

**FIGURE 2:**
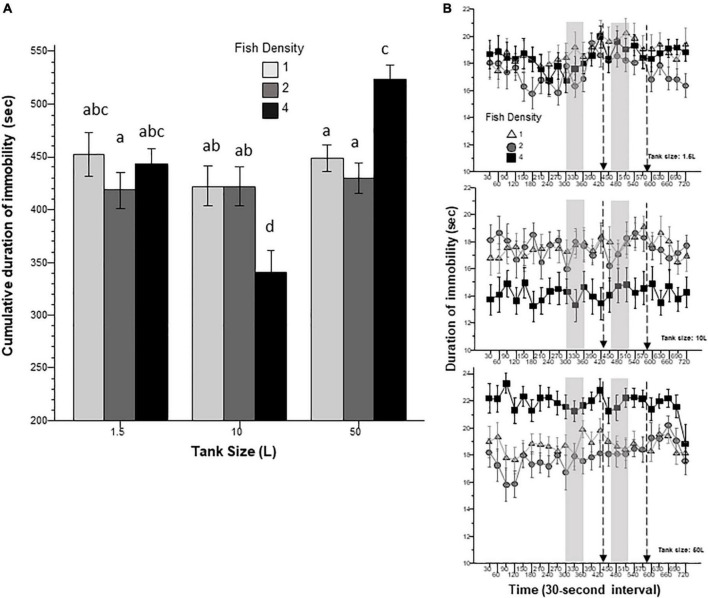
Speed is affected by prior fish density and home tank size. Panel **(A)** shows the average speed exhibited by zebrafish during the entire 12 min behavioral test period. The size of the housing tank is shown on the *X*-axis and the fish density is shown by the legend. Bars marked by at least one common letter represent groups that are not different significantly (*p* > 0.05). Note that although individual differences were not found among groups, ANOVA revealed a significant fish density x tank size interaction. Panel **(B)** shows the speed of experimental zebrafish as a function of 30-s intervals. The upper line-graph shows the performance of fish housed in 1.5 L tanks, the middle line-graph the performance of fish housed in 10 L tanks and the lower line-graph the performance of fish housed in 50 L tanks. The fish density of fish is shown by the legend. The vertical semi-transparent gray bars indicate the period during which still images of zebrafish were shown on the computer screen, and the broken lines with the arrowhead indicate the short period of administering a computer animated expanding dot. Mean + S.E.M. are shown. For detailed statistical results, see Table 1 and the section “Results.”

Swimming speed may increase as a result of exploration of a novel environment or when the fish are attempting to actively escape an aversive context or cue. Speed, although may be assumed to be negatively correlated with immobility duration, has been shown to represent an independent measure of behavior in zebrafish (Blaser and Gerlai, 2006; Kalueff et al., 2013; Abozaid et al., 2020). The results shown in Figure 2 suggest that speed also varied according to the prior housing conditions, but the pattern of group differences was unlike those seen in immobility duration. Although ANOVA found no significant main effects of fish density and housing tank size, it did reveal a significant interaction between these two factors. Despite the significant interaction term identified by ANOVA Tukey’s HSD *post-hoc* test did not detect any group differences. Nevertheless, Figure 2A shows an apparent decrease of speed with fish density in the 1.5 L tank, an increased speed with fish density in the 50 L tank, and a lack of change in the 10 L tank, pattern of results that confirm the identified fish density x tank size interaction. Variance Analysis of the 30-s interval data revealed a significant interval effect, but as in the case of immobility duration, temporal changes in speed were found independent of housing conditions, i.e., no significant interactions between interval and fish density or tank size were found for this behavior.

**FIGURE 3:**
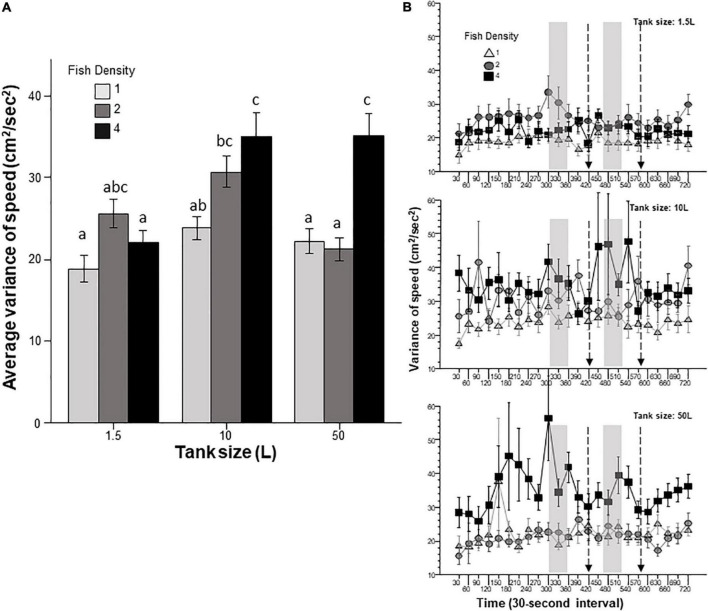
Intra-individual variance of speed is affected by prior fish density and home tank size. Panel **(A)** shows the average variance of speed exhibited by zebrafish during the entire 12 min behavioral test period. The size of the housing tank is shown on the *X*-axis and the fish density is shown by the legend. Bars marked by at least one common letter represent groups that are not different significantly (*p* > 0.05). Panel **(B)** shows the variance of speed of experimental zebrafish as a function of 30-s intervals. The upper line-graph shows the performance of fish housed in 1.5 L tanks, the middle line-graph the performance of fish housed in 10 L tanks and the lower line-graph the performance of fish housed in 50 L tanks. The fish density of fish is shown by the legend. The vertical semi-transparent gray bars indicate the period during which still images of zebrafish were shown on the computer screen, and the broken lines with the arrowhead indicate the short period of administering a computer animated expanding dot. Mean + S.E.M. are shown. For detailed statistical results, see Table 1 and the section “Results.”

Intra-individual temporal variance of speed (Figure 3) measures whether the experimental zebrafish swims with a consistent speed (low variance) or whether it changes its swim speed a lot (high variance). Consistent swim speed (low intra-individual variance) often characterizes a habituated zebrafish familiar with its environment. Variance Analysis of the variance of swim speed showed that both fish density and housing tank size had a highly significant effect. Furthermore, these effects were found non-additive as a significant interaction between these factors was also revealed. Tukey’s HSD *post-hoc* multiple comparison test confirmed the ANOVA results and revealed several significant group differences (Figure 3A) and suggested that fish housed at the highest density in the larger tanks varied their speed most. The effect of interval was found significant, but interval did not significantly interact with fish density or tank size.

**FIGURE 4:**
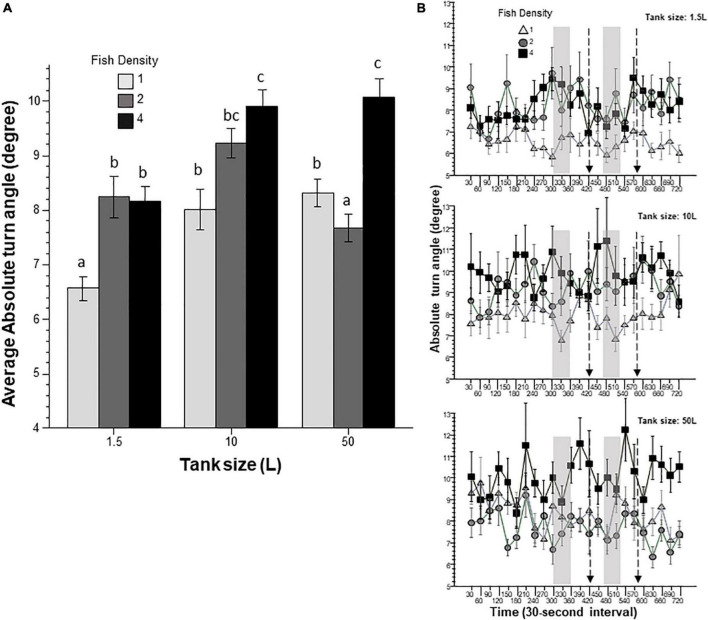
Absolute turn angle is affected by prior fish density and home tank size. Panel **(A)** shows average turn angle exhibited by zebrafish during the entire 12 min behavioral test period. The size of the housing tank is shown on the *X*-axis and the fish density is shown by the legend. Bars marked by at least one common letter represent groups that are not different significantly (*p* > 0.05). Panel **(B)** shows turn angle of experimental zebrafish as a function of 30-s intervals. The upper line-graph shows the performance of fish housed in 1.5 L tanks, the middle line-graph the performance of fish housed in 10 L tanks and the lower line-graph the performance of fish housed in 50 L tanks. The fish density of fish is shown by the legend. The vertical semi-transparent gray bars indicate the period during which still images of zebrafish were shown on the computer screen, and the broken lines with the arrowhead indicate the short period of administering a computer animated expanding dot. Mean + S.E.M. are shown. For detailed statistical results, see Table 1 and the section “Results.”

Absolute turn angle (Figure 4) quantifies the amount of turning irrespective of its direction. Turning has been found to correlate with a variety of behavioral responses, strategies, or states, including anxiety (erratic movement) as well as exploratory behavior. When zebrafish are introduced to a novel environment they increase their turning (Kalueff et al., 2013; Gerlai and Tran, 2016). Here, absolute turn angle was found to be significantly affected by prior housing conditions, including tank size and fish density. Furthermore, ANOVA also revealed a significant interaction between these two factors. Tukey’s HSD *post-hoc* multiple comparison test identified numerous group differences (Figure 4A) and suggested that fish housed at higher densities in larger tanks turn more. On the other hand, interval was found not to have any significant effect and it also did not significantly interact with fish density and housing tank size.

**FIGURE 5:**
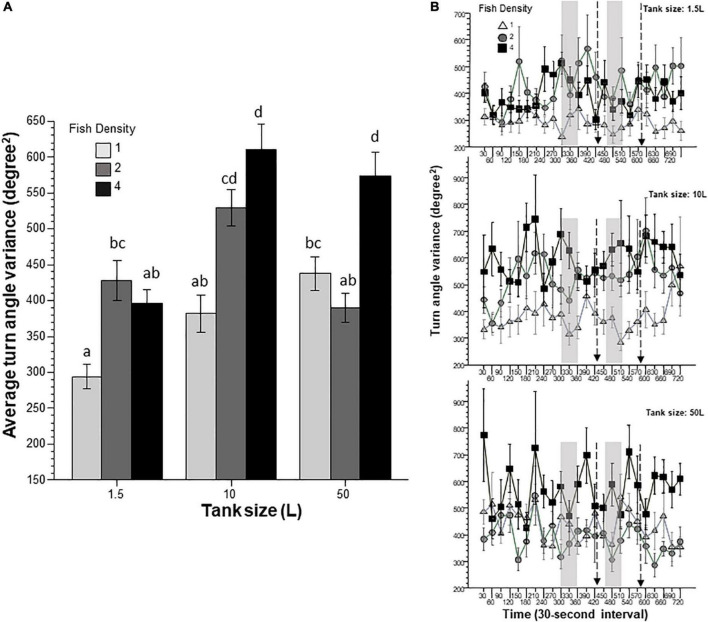
Intra-individual variance of absolute turn angle is affected by prior fish density and home tank size. Panel **(A)** shows average turn angle variance exhibited by zebrafish during the entire 12 min behavioral test period. The size of the housing tank is shown on the *X*-axis and the fish density is shown by the legend. Bars marked by at least one common letter represent groups that are not different significantly (*p* > 0.05). Panel **(B)** shows turn angle variance of experimental zebrafish as a function of 30-s intervals. The upper line-graph shows the performance of fish housed in 1.5 L tanks, the middle line-graph the performance of fish housed in 10 L tanks and the lower line-graph the performance of fish housed in 50 L tanks. The fish density of fish is shown by the legend. The vertical semi-transparent gray bars indicate the period during which still images of zebrafish were shown on the computer screen, and the broken lines with the arrowhead indicate the short period of administering a computer animated expanding dot. Mean + S.E.M. are shown. For detailed statistical results, see Table 1 and the section “Results.”

Intra-individual variance of absolute turn angle (Figure 5) quantifies how consistently/inconsistently a zebrafish turns. Turn angle variance tends to increase in novel environments, and may be used as a measure of fear or anxiety, but it is also expected to increase when the non-anxious fish actively explores its surroundings (Kalueff et al., 2013; Gerlai and Tran, 2016). Here, turn angle variance was found to be significantly affected by prior housing conditions including tank size and fish density. Furthermore, a significant interaction between these two factors was also found. Tukey’s HSD *post-hoc* multiple comparison test identified numerous differences among groups suggesting that fish housed at the highest density vary their turning most if previously they were housed in larger tanks. On the other hand, the effect of interval was non-significant and the interactions between interval and the other two factors were also non-significant.

**FIGURE 6:**
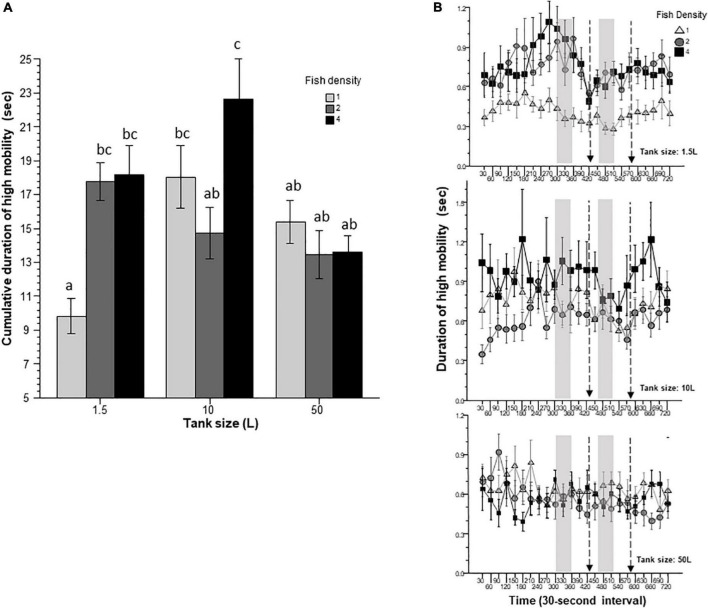
Duration of high mobility is affected by prior fish density and home tank size. Panel **(A)** shows cumulative duration of high mobility exhibited by zebrafish during the entire 12 min behavioral test period. The size of the housing tank is shown on the *X*-axis and the fish density is shown by the legend. Bars marked by at least one common letter represent groups that are not different significantly (*p* > 0.05). Panel **(B)** shows duration of high mobility of experimental zebrafish as a function of 30-s intervals. The upper line-graph shows the performance of fish housed in 1.5 L tanks, the middle line-graph the performance of fish housed in 10 L tanks and the lower line-graph the performance of fish housed in 50 L tanks. The fish density of fish is shown by the legend. The vertical semi-transparent gray bars indicate the period during which still images of zebrafish were shown on the computer screen, and the broken lines with the arrowhead indicate the short period of administering a computer animated expanding dot. Mean + S.E.M. are shown. For detailed statistical results, see Table 1 and the section “Results.”

Duration of high mobility may reflect escape responses from aversive contexts and/or cues. For example, leaping (fast and short movement in one direction) or erratic movement (fast zig-zagging) are associated with elevated swim speed, and are seen in zebrafish under aversive conditions (Kalueff et al., 2013). Duration of high mobility (Figure 6) was intended to capture/quantify such events. Duration of high mobility was found by ANOVA to be significantly affected by prior housing conditions, including housing tank size and fish density. Also, these two factors were found to significantly interact. Tukey’s HSD *post-hoc* multiple comparison test revealed numerous group differences. The results suggested that fish housed in the largest tank and those housed alone in the smallest tank exhibited the shortest duration of high mobility. ANOVA also found interval to have a significant effect, but the interaction terms between interval and fish density or tank size were found non-significant.

**FIGURE 7:**
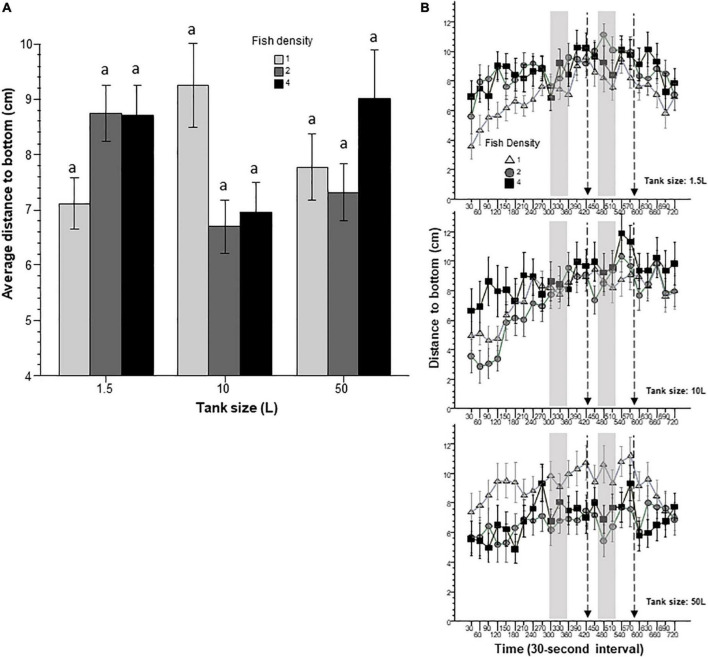
Distance to bottom is affected by prior housing conditions. Panel **(A)** shows the average distance the fish were from the bottom during the entire 12 min behavioral test period. The size of the housing tank is shown on the *X*-axis and the fish density is shown by the legend. Bars marked by at least one common letter represent groups that are not different significantly (*p* > 0.05). Note the lack of significant differences among any groups found by Tukey’s HSD test despite the significant fish density × housing tank size interaction revealed by ANOVA. Panel **(B)** shows the distance zebrafish were from the bottom as a function of 30-s intervals. The upper line-graph shows the performance of fish housed in 1.5 L tanks, the middle line-graph the performance of fish housed in 10 L tanks and the lower line-graph the performance of fish housed in 50 L tanks. The fish density of fish is shown by the legend. The vertical semi-transparent gray bars indicate the period during which still images of zebrafish were shown on the computer screen, and the broken lines with the arrowhead indicate the short period of administering a computer animated expanding dot. Mean + S.E.M. are shown. For detailed statistical results, see Table 1 and the section “Results.”

Bottom dwelling, or diving, has been argued to be a sign of fear/anxiety in zebrafish. To quantify this response, we measured how far the fish swam from the bottom of the experimental tank, i.e., the distance to bottom (Figure 7). Although ANOVA found no significant effects of fish density and housing tank size, the interaction between these two factors was significant. Although Tukey’s HSD *post-hoc* test revealed no significant differences between any of the groups, the pattern of results shown on Figure 7A offers an explanation for the significant interaction term: fish housed at higher densities in the 1.5 L tank increased their distance to bottom whereas fish housed at higher densities in the 10 L tank decreased it, compared to fish housed at the lowest density. Variance Analysis of the 30-s time resolution data revealed a significant interval effect. Perusal of Figure 7B suggests that fish initially stayed close to the bottom, and as the behavioral recording session progressed started to move further away from the bottom. ANOVA also revealed a significant housing tank density x interval interaction, which is likely the result of fish of the lowest density groups showing the most robust temporal change.

**FIGURE 8:**
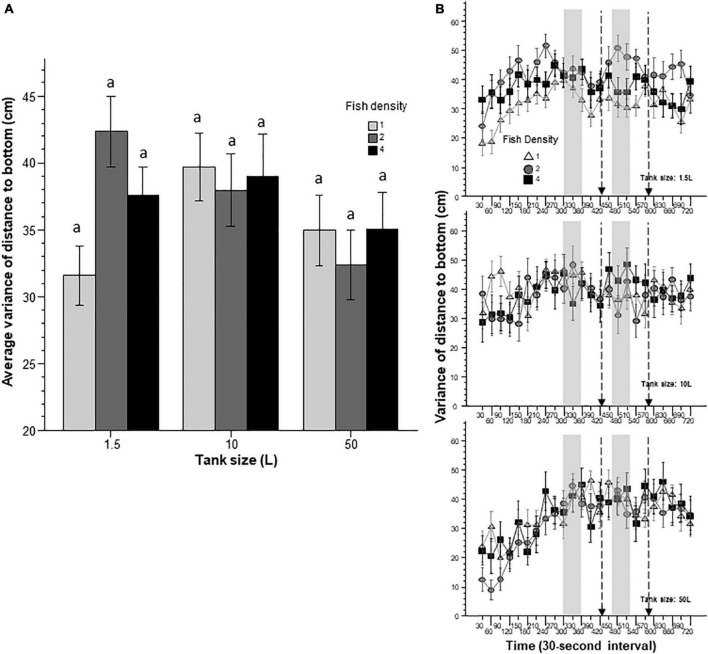
Intra-individual temporal variance of distance to bottom appears to be affected by prior housing conditions. Panel **(A)** shows the average variance of distance the fish were from the bottom during the entire 12 min behavioral test period. The size of the housing tank is shown on the *X*-axis and the fish density is shown by the legend. Bars marked by at least one common letter represent groups that are not different significantly (*p* > 0.05). Note the lack of significant differences among any groups found by Tukey’s HSD and that tank size effect and the tank size × fish density interaction was found by ANOVA to border, but did not reach, the level of significance. Panel **(B)** shows the variance of distance zebrafish were from the bottom as a function of 30-s intervals. The upper line-graph shows the performance of fish housed in 1.5 L tanks, the middle line-graph the performance of fish housed in 10 L tanks and the lower line-graph the performance of fish housed in 50 L tanks. The fish density of fish is shown by the legend. The vertical semi-transparent gray bars indicate the period during which still images of zebrafish were shown on the computer screen, and the broken lines with the arrowhead indicate the short period of administering a computer animated expanding dot. Mean + S.E.M. are shown. For detailed statistical results, see Table 1 and the section “Results.”

In addition to the distance the experimental fish swam from the bottom, we also measured the intra-individual variance of distance to bottom. This behavioral parameter measures how consistently (low variance) or inconsistently (high variance) the fish positioned itself relative to the bottom, a measure of vertical exploration. Vertical exploration has been found to decrease in response to novelty and aversive stimuli (Kalueff et al., 2013; Gerlai and Tran, 2016). Here, as before, we found vertical exploration to generally increase with time (Figure 8B). Supporting this observation was the significant interval effect found by ANOVA. However, this increase was not uniform across all groups, as shown by the significant interval x tank size interaction. Further analysis of the data pooled (averaged) for the entire behavioral recording session showed that the effect of tank size bordered but did not reach significance. Similarly, the fish density x tank size interaction bordered but did not reach significance.

**FIGURE 9:**
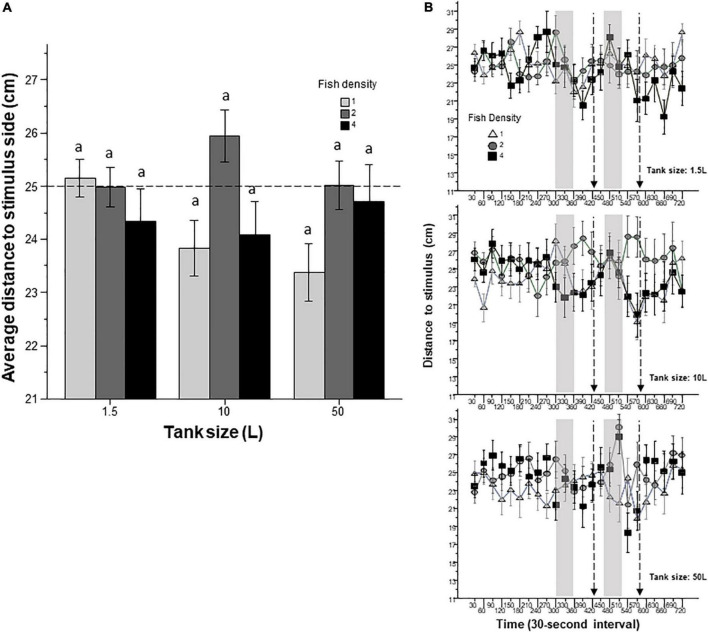
Distance to stimulus screen is affected by prior fish density. Panel **(A)** shows the average distance the fish were from the stimulus screen during the entire 12 min behavioral test period. The size of the housing tank is shown on the *X*-axis and the fish density is shown by the legend. Bars marked by at least one common letter represent groups that are not different significantly (*p* > 0.05). Note the lack of significant differences among any groups found by Tukey’s HSD test despite the significant fish density effect revealed by ANOVA. Panel **(B)** shows the distance zebrafish were from the stimulus screen as a function of 30-s intervals. The upper line-graph shows the performance of fish housed in 1.5 L tanks, the middle line-graph the performance of fish housed in 10 L tanks and the lower line-graph the performance of fish housed in 50 L tanks. The fish density of fish is shown by the legend. The vertical semi-transparent gray bars indicate the period during which still images of zebrafish were shown on the computer screen, and the broken lines with the arrowhead indicate the short periods of administering a computer animated expanding dot. Mean + S.E.M. are shown. For detailed statistical results, see Table 1 and the section “Results.”

To mimic the natural environment, for example the appearance of shoal mates, still images of zebrafish were presented for 1 min twice during the behavioral session. An expanding black filled circle, mimicking a rapidly approaching fish predator was also presented for a few seconds twice to mimic an approaching fish predator. Distance to the stimulus side, i.e., to the computer monitor that presented the given visual stimulus, was measured throughout the behavioral recording session. Prior housing conditions appeared to have some effect on this behavioral measure (Figure 9). ANOVA found the effect of fish density significant, but tank size and the tank size x fish density interaction were non-significant. As expected, interval had a significant effect, and the interval × fish density interaction was also significant. To further examine this latter finding, and because we expected stimulus-specific temporal changes in the distance to stimulus side, we calculated the difference between the average of the distance the fish were from the stimulus screen during the first 5 min (habituation period) and the average of the distance the fish were from the stimulus during the 30 s intervals when the stimulus was being shown. This calculation was performed separately for the shoaling stimulus (conspecific images) and for the expanding black circle. ANOVA found that prior housing conditions had no significant effect on how the zebrafish responded to the shoaling stimulus. However, ANOVA did find a significant effect of fish density on how the fish responded to the expanding circle, suggesting that fish housed in medium density increased their distance to, i.e., avoided the expanding circle more than fish in the other groups. Nevertheless, subsequent *post-hoc* Tukey’s HSD test found no significant differences among the nine experimental groups in this behavioral measure.

To better understand how certain behaviors correlated with each other and with the treatment conditions, housing tank size and fish density, we calculated Pearson product moment correlation coefficients and subjected the correlation matrix to Principal Component Analysis using Varimax rotation with Kaiser normalization. The rotation converged after five iterations, and the analysis extracted four principal components with eigen values reaching or exceeding 1 (Table 3). The cumulative variance explained by these four principal components was 68% of total variance found in the data with the 1st, 2nd, 3rd, and 4th principal component each explaining 28.2, 16.7, 11.5, and 11.3% of the total variance, respectively.

**TABLE 3:** Principal component analysis: Rotated component matrix shows correlation groups of behaviors and treatment factors.

	Principal component			
	1	2	3	4
Fish density	0.602			0.216
Tank size	0.291	–0.389	0.231	
Immobility (cumulative duration)			–0.893	
Speed (average)		0.816		0.231
Variance of speed (average)	0.562	0.578		0.236
Absolute turn angle (average)	0.941			
Variance of turn angle (average)	0.931			
High mobility (cumulative duration)	0.412	0.703		
Distance to bottom (average)				0.857
Variance of distance to bottom (average)		0.261		0.665
Change of distance to shoal (average)				0.799
Change of distance to dot (average)				0.804

The data matrix shows the four principal components whose eigen values were at least 1. The matrix shows major component loadings, i.e., loadings whose absolute value was larger than 0.20. Loadings without a sign are positive, loadings with the negative sign are negative. The loadings are essentially bivariate correlation coefficients between the given principal component and the corresponding behavior or treatment factor. Also, variables with loadings with the same sign listed under the same principal component indicate positive correlation between the variables. Variables with loadings with different signs listed under the same principal component are negatively correlated. Note that the first principal component contains large loadings of fish density and tank size along with several behaviors with the same sign (positive) and that these behaviors represent change in speed or direction of movement. Also note that the rest of the principal components contain major loadings of only one or the other treatment factor along with numerous behaviors. For further details of results and their interpretation see the sections “Results and Discussion.”

The first principal component extracted includes major loadings of both treatment conditions, fish density and tank size along with behavioral variables that all measure some aspects of change in the direction or the speed of movement. Finding the two treatment conditions to load on the same factor with the same (positive) sign along with these behavioral parameters demonstrates that both fish density and tank size affected these behaviors. Principal components 2, 3, and 4 have major loading of either fish density or tank size, along with some behaviors. This result demonstrates that fish density and tank size separately and independently affected the corresponding behaviors.

## Discussion

In this study, we exposed zebrafish for 2 weeks to different housing conditions, i.e., housing fish densities and tank sizes, and tested the potential effects of this treatment in a short behavioral test by placing the experimental fish singly in a novel tank. In addition, to mimic the natural environment, we also provided visual stimuli on computer screens flanking the test tank at specific periods during the behavioral test session. Handling and novelty itself are aversive and induce fear and anxiety-like responses in zebrafish (Blaser and Rosemberg, 2012; Kalueff et al., 2013; Gerlai and Tran, 2016), and we expected to see housing condition dependent effects on these behavioral responses. Analysis of the swim path parameters of the experimental fish confirmed numerous significant housing condition dependent changes. For several behaviors, the results demonstrated that housing fish density and housing tank size induced changes that were significant but non-additive. That is, the effect of fish density depended upon the tank size in which the fish were housed, or vice versa, the effect of tank size depended upon at what density the experimental fish were housed. For example, duration of immobility was increased by prior housing of fish at high density if they were housed in the 50 L tank, and decreased if they were housed in the 10 L tank, but was unaffected if they were in the 1.5 L tank. Intra-individual variance of speed, absolute turn angle and intra-individual variance of turn angle were all increased by prior high-density housing in both the 50 L and the 10 L tanks but not if the fish were housed in the 1.5 L tank. High mobility in the behavioral test was also affected by prior housing conditions. Fish housed in the 1.5 and 10 L tanks exhibited fish density dependent changes in high mobility, but fish housed in the 50 L tank did not. Unexpectedly, analysis of swim speed and the distance measures, including distance to bottom, intra-individual variance of distance to bottom (vertical exploration) and distance to stimulus yielded inconsistent results that are difficult to interpret. While ANOVA detected significant or close to significant results for main effects or the interaction term for these behaviors, and although the pattern of effects depicted on Figures 2, 7–9 also suggests potential housing conditions dependent changes, Tukey’s HSD *post-hoc* tests found all group differences non-significant. This was unexpected because these behavioral measures (swim speed, distance to bottom, vertical exploration) are often claimed to be reliable indicators of fear or anxiety in zebrafish (Cachat et al., 2010; Blaser and Rosemberg, 2012; Kalueff et al., 2013; Gerlai and Tran, 2016). Similarly, Tukey’s HSD *post-hoc* tests, failed to detect any significant housing conditions related group differences in how the experimental zebrafish changed their distance to the stimulus side in response to the presentation of the visual stimuli applied.

Given the large number of often complex and behavior specific changes induced by housing conditions in the nine groups of experimental zebrafish, we conducted a multivariate analysis to explore how the behavioral changes induced by housing conditions may relate to each other using Principal Component Analysis (PCA). The PCA Rotated component loading matrix in which input variables are organized into correlation groups (the principal components) revealed four independent (orthogonal or non-correlating) principal components (Table 3). The first principal component had major loadings of both treatment conditions (fish density and tank size) along with behavioral measures (variance of speed, turning, variance of turning and high mobility) that share one common feature: they all quantify aspects of changes in the direction or speed of movement. The positive signs of all loadings on this principal component suggest that keeping fish at higher density and in larger tanks for 2 weeks both resulted in increased variability in the direction and speed of swimming in zebrafish tested in the novel test tank. Principal components 2, 3, and 4, however, showed another interesting result. These components only contained major loadings of either tank size or fish density but not both. This result suggests that fish density and tank size had independent effects on some behaviors. Principal component 2 had major loadings of tank size and behaviors including speed, variance of speed, high mobility, and variance of distance to bottom. What is common to all these behaviors is that they all measure aspects of active/fast locomotion: speed quantifies velocity, variance of speed the intra-individual temporal variability of velocity, high mobility the duration of fast swimming episodes, and variance of distance to bottom, the movement along the vertical axis, i.e., vertical exploration. Importantly, the sign of loading of tank size was negative while the sign of loadings for these behaviors was positive on principal component 2, demonstrating that fish that were exposed to smaller tank sizes for 2 weeks exhibited more rapid swimming responses in the novel tank test. Principal component three had major negative loading of tank size and the two distance measures (change of distance to shoal image and to the expanding dot) and a major loading with negative sign for immobility. This is also a notable result for several reasons. One, unlike we assumed, the change of distance to the expanding dot and to the shoal image positively correlated. That is, fish that swam further away from the stimulus screen when the expanding dot was being shown (compared to their distance prior to the delivery of the stimulus), also swam further away in response to the shoaling image and also exhibited less immobility (and vice versa). This result unexpectedly suggests that both the expanding dot and the image of conspecifics was aversive. The latter is likely because unlike animated (moving) conspecific images (Qin et al., 2014), motionless conspecific images mimic freezing, i.e., immobile zebrafish, which may be aversive for the observing experimental zebrafish. Immobility (or freezing) is often regarded as one of the most reliable measures of strong fear and anxiety in zebrafish (Egan et al., 2009; Cachat et al., 2010; Blaser and Rosemberg, 2012; Kalueff et al., 2013; Gerlai and Tran, 2016). Increased distance to the visual stimuli coupled with decreased freezing may represent exploration of the tank, perhaps an active avoidance reaction to the stimuli. The loadings of principal component three also demonstrate that fish that were exposed to larger housing tanks stayed immobile less and increased their distance to the presented images more, i.e., likely chose an active rather than passive avoidance strategy. As freezing or immobility is considered one of the strongest fear/anxiety responses in zebrafish, the active avoidance/increased exploratory responses resulting from keeping the fish in larger housing tanks suggest anxiolytic effects of this housing method. The last principal component, principal component four, had major loadings of fish density, speed measures and particularly large loadings of the behaviors distance to bottom and variance of distance to bottom with the same sign. This suggests that fish that were housed at higher densities swam faster and further away from the bottom, and changed their speed and their distance to bottom more than fish that were housed at lower densities. As higher swimming activity, increased distance from bottom and increased vertical exploration have all been thought to be associated with reduced fear and anxiety (Blaser and Rosemberg, 2012; Kalueff et al., 2013; Gerlai, 2020), this principal component thus suggests that prior housing of fish in higher densities may have fear/anxiety reducing effects when tested subsequently in a novel tank task. In summary, overall, our results imply that keeping the zebrafish in larger tanks and at higher densities may be beneficial.

One potential limitation of our study is lack of replicates, i.e., the fact that we ran a single experiment without at least one repetition. Replicates can be thought of as temporal replicates, i.e., the same experiment conducted multiple times at different time points, months or seasons, for example. We argue that this is unlikely to be a limitation in the current study because the environmental parameters (including water parameters, humidity, temperature, air pressure, light cycle, feeding cycle) are all precisely controlled and are independent of seasonal or other temporal variation in the Gerlai Zebrafish Facility. However, one can argue that lack of replicates poses a problem analogous to the “litter effect,” a problem that remains a topic of concern even in the analysis of the most well studied biomedical research organism, the house mouse (Jiménez and Zylka, 2021). Essentially, this confounding problem occurs when one uses a given litter of mice for one treatment group and another litter for another treatment group. The litter effect is due to potential genetic differences (different parents), and/or to cage/litter specific conditions (random variation in food/water access, or in level of social interaction resulting, for example, from different number of pups in each litter). We argue, however, that such litter-effect-like problems are unlikely to occur in our study with zebrafish, given that unlike mice, zebrafish produce 2–300 offspring per female, there is no parental care, and the fertilized eggs we generated from multiple spawnings among several males and females were pooled, hatched together and the fry were raised together under identical conditions. Furthermore, we selected our experimental fish randomly from this pool, i.e., from thousands of these offspring. Nevertheless, yet another reason for the need for replicates still remains: tank specific effects. Just like cage-specific effects in case of mice, tank specific effects for zebrafish may be a confound. For example, a given tank may receive a slightly less strong water flow and thus fish may be exposed to higher levels of organic waste products and reduced levels of oxygen. Or, another tank may be further way from a ceiling mounted fluorescent light fixture and thus may receive less illumination. Such tank specific effects may represent a confound especially if large number of subjects are tested from a single tank representing a particular treatment condition. Thus, from a statistical analysis perspective, one could argue that the tank average and not the data point of the individual fish should be considered the unit of analysis when one calculates the sample size. This is a valid argument. However, it is practically difficult to address, as it would have required us, for example, to have twenty 50 L tanks for each of the fish densities, and use a single subject from each of these tanks. Another problem with the latter experimental design, is that it will inflate experimental error variation. Because of the large number of experimental treatment tanks required for this design, such tanks would have had to be placed on a large number of separate Aquaneering system racks, i.e., would have received their own separate filtration and spatial location placement in the facility. Given that equalizing filtration efficiency and setting up exactly the same water parameters with exactly the same organic waste products and equalizing space specific environmental stimuli are all practically impossible, the Aquaneering rack specific environmental conditions would have created increased error variation. Instead, our experimental design allowed us to place all treatment tanks (the different volumes of tanks housing different number of fish) on the same, or on limited number of, system racks, and near or next to each other, which allowed us to provide water to these tanks from the same sump/filtration system and to provide the same or very similar environmental stimuli, including level of illumination.

The last point we wish to emphasize is another reason why we consider our study only as proof-of-concept analysis, one which should persuade others to conduct further and more detailed analyses. Although the three housing tank sizes and three fish densities employed allowed us to systematically analyze the effects of these housing conditions, the small number of levels of these factors did not allow us to explore a wide enough range of conditions and their potential effects. Notably, zebrafish are routinely kept at more extremely crowded conditions and at widely varying tank sizes. For example, the Association for Assessment and Accreditation of Laboratory Animal Care International (AAALAC International, headquartered in Frederick, MD, USA) recommends five adult zebrafish per litre,^1^ a density higher than the highest employed in the current study, but lower than what some facilities employ. We argue that only when a wider range of conditions are explored, one can conclude about what may be the most optimal way to house the zebrafish. Although our current study did not attempt to use such extreme fish densities as recommended and employed by others, it already demonstrated that housing zebrafish for only 2 weeks under distinct conditions at different fish densities and in differently sized holding tanks does have measurable, significant, effects on the behavior of zebrafish. Ascertaining whether the behavioral changes we observed here represent modifications in fear, anxiety or stress will require a battery of behavioral tests specifically designed to assess such responses along with physiological and neurobiological measures, including analysis of cortisol and neurochemical responses. Nevertheless, the current results have already demonstrated that tank size and fish density can act in concert, i.e., in an interactive way, as well as independently, and can significantly affect a variety of behavioral responses.

## Data availability statement

The raw data supporting the conclusions of this article will be made available by the authors, without undue reservation.

## Ethics statement

This animal study was reviewed and approved by the Local Animal Care Committee of University of Toronto Mississauga.

## Author contributions

RG conceived and directed the study, performed the statistical analysis, and interpreted the results. RG and BT planned the study. SS conducted the experiments. SS, BT, and RG wrote the manuscript. All authors contributed to the article and approved the submitted version.

## Footnotes

1
https://www.aaalac.org/pub/?id=E9019693-90EC-FC4A-526E-E8236CC13B28
